# The Protection of Salidroside of the Heart against Acute Exhaustive Injury and Molecular Mechanism in Rat

**DOI:** 10.1155/2013/507832

**Published:** 2013-12-18

**Authors:** Yunru Wang, Peng Xu, Yang Wang, Haiyan Liu, Yuwen Zhou, Xuebin Cao

**Affiliations:** Department of Cardiology, Geriatric Cardiovascular Disease Research and Treatment Center, No. 252 Hospital of PLA, Baoding 071000, China

## Abstract

*Objective*. To investigate the protection of salidroside of the heart against acute exhaustive injury and its mechanism of antioxidative stress and MAPKs signal transduction. *Method*. Adult male SD rats were divided into four groups randomly. Cardiomyocytes ultrastructure was observed by optical microscopy and transmission electron microscopy. The contents of CK, CK-MB, LDH, MDA, and SOD were determined by ELISA method, and the phosphorylation degrees of ERK and p38 MAPK were assayed by Western blotting. Cardiac function of isolated rat heart ischemia/reperfusion was detected by Langendorff technique. *Results*. Salidroside reduced the myocardium ultrastructure injury caused by exhaustive swimming, decreased the contents of CK, CK-MB, and LDH, improved the LVDP, ±LV *dp*/*dt*_max_ under the basic condition, reduced the content of MDA and the phosphorylation degree of p38 MAPK, and increased the content of SOD and the phosphorylation degree of ERK in acute exhaustive rats. *Conclusion*. Salidroside has the protection of the heart against acute exhaustive injury. The cardioprotection is mainly mediated by antioxidative stress and MAPKs signal transduction through reducing the content of MDA, increasing the content of SOD, and increasing p-ERK and decreasing p-p38 protein expressions in rat myocardium, which might be the mechanisms of the cardioprotective effect of salidroside.

## 1. Introduction

Exhaustive exercise is a pathological state of multiple organ dysfunction due to the strong and durable exercise load which is beyond the bearing ability of the body. It will increase the oxygen consumption of cardiomyocytes, which may produce an imbalance between reactive oxygen species (ROS) and antioxidants, inducing oxidative stress as a result of increased ROS production [1–3] and damage to cardiac structure, metabolism, and function [4, 5]. More specifically, oxidative stress is the imbalance between oxidation and antioxidation system. Superoxide dismutase (SOD) is one of the important enzymes to eliminate ROS and malondialdehyde (MDA) is the terminal product of the membrane lipid peroxidation; the changes of their contents can reflect the degree of oxidative stress of cardiomyocytes.

Exhaustive exercise can destroy the balance of antagonism between atrial natriuretic peptide and endothelin, which will cause sustained contraction of coronary artery, making the blood supply of coronary artery unable to satisfy the demand of cardiomyocytes for blood and oxygen and then induce continuous ischemia-hypoxia of myocardium and the damage factor will increase at the same time; both of them are deleterious to myocardium [6, 7]. Mitogen-activated protein kinases (MAPKs) are one of the important signal transduction systems in cell, including the ways of extra cellular signal-regulated kinase (ERK) and p38 MAPK. Research shows that the activation of ERK can inhibit the apoptosis of myocardial cell and p38 MAPK might promote the apoptosis of cardiomyocytes [8–11]. As an important second messenger in the cell, ROS also can mediate cell survival and death by influencing the ways of ERK and p38 MAPK.

Salidroside (SAL) as one of the effective components of *Rhodiola rosea* L. has the protection against the liver and kidney injury of rat, and it is related to the function of antioxidative stress and antiapoptosis [12, 13]. In addition, salidroside has good protection against acute myocardial ischemia injury and isolated heart ischemia/reperfusion injury of normal rats. It can increase myocardial contraction force, improve myocardial ischemia, and reduce myocardial ischemia infarction area [14, 15]. However, there is no research regarding whether salidroside is protective against acute myocardial ischemia injury caused by exhaustion exercise and exhaustive heart ischemia-reperfusion injury at present. Thus, in this study, we established the model of isolated rat heart ischemia/reperfusion by Langendorff technique to investigate the effect of salidroside on cardiac function of acute exhaustion rat and its changes after ischemia/reperfusion and discussed whether the protection mechanisms are antioxidation stress and MAPKs signal transduction.

## 2. Method

### 2.1. Material

99% salidroside powder (Lot: 080901-1) was purchased from Ying Xuan Biochemicals Co. Ltd. (Shanghai, China); ELISA kits were obtained from BD Co. (New York, USA); antibodies used were as follows: total p38 MAPK (D13E1) 1 : 1000, phospho-p38 MAPK (Thr180/Tyr182) (D3F9) 1 : 1000, total SAPK/JNK 1 : 1000, phospho-SAPK/JNK (Thr183/Tyr185) 1 : 1000, total p44/42 MAPK (Erk1/2) (137F5) 1 : 2000, and phosphor-p44/42 MAPK (Erk1/2) (Thr202/Tyr204) 1 : 2000 were all purchased from CST Co. (USA). Horseradish peroxidase labeling goat anti-mouse IgG was obtained from CWBIO Co. Ltd. (Beijing, China). All of the chemicals were of analytical reagent grade.

Forty adult male Sprague Dawley (SD) rats of pathogen-free were provided by Laboratory Animal Center of the Academy of Military Medical Sciences (Beijing), certification number SCXK (Beijing): 2003-1-003. The animal experimental procedures were approved by the university committee for animal experiments and in accordance with the principles outlined in the NIH Guide for the Care and Use of Laboratory Animals.

A rat isolated perfusion system (120102EZ-220, Radnoti Co., USA), a rat physiological recorder system (ML880/P16, AD Co. USA), a microplate reader (Thermo fisher SC, FI), a biochemical analyzer (7600-02, HITACHI, Japan), a transmission electron microscope (TEM) (H-7500, HITACHI, Japan), and an ultramicrotome (Leica instrument Co. Ltd., GER) were used in the experiments.

### 2.2. The Establishment of Exhaustive Animal Model

The classical Thomas method [16] was used to establish the model of acute exhaustive cardiac injury through exhaustive swimming. The rats were divided into control group (Con), salidroside group (Sal), salidroside-acute exhaustive swimming group (SE), and acute exhaustive swimming group (EE) randomly and averagely. The rats of Con group and SE group were given 0.9% NaCl (12 mL·kg^−1^·d^−1^) by intraperitoneal injection for fourteen days; meanwhile the rats of Sal group and SE group were given salidroside (24 mg·kg^−1^·d^−1^) in the same way. Then the rats of SE group and EE group submitted to one-time exhaustive swimming, stopped the exhaustive exercise when met the Thomas standard of exhaustion and dried the fur to avoid the rats sick.

### 2.3. The Collection of Serum

Blood was collected from abdominal aorta by plain tube within 2 h after exhausted exercise and centrifuged (3000 g/min) for twenty minutes. The serum was preserved at −80°C.

### 2.4. The Protein Extraction of Myocardium

Freeze the rat heart immediately after the heart was taken out and washed in PSB solution. Then cut the left ventricular myocardial into small pieces, weighed 0.05 g, and put them into the extraction reagent, which consisted of 3.6 mL of lysis buffer, 400 *μ*L of PMSF, homogenized and lysed for thirty minutes and then centrifuged (10000 g/min) for five minutes at 4°C. The supernatant was preserved at −20°C until needed.

### 2.5. The Preparation of Myocardial Specimen

The rat heart was fixed in 10% formaldehyde and preserved at normal temperature. Then observe it by optical microscope after HE coloration.

A small piece (2 mm × 1 mm × 1 mm) of subendocardial myocardium from the root of left ventricular papillary muscle was taken and fixed in 0.1 mmol/L phosphate buffer (pH = 7.2) which included 3% glutaraldehyde and 1.5% paraformaldehyde at 4°C. Then cut it into small pieces of 1 mm^3^ and continue fixed in the above solution for 4 h. Fixed in 1% osmic acid again at 4°C for 1.5 h after rinsed by phosphate buffer. Afterwards, the tissue was dehydrated by alcohol and acetone in order, embedded by epoxy resin 618, located by semithin sectioning, and sliced into ultrathin sections in a thickness of 60 nm. The sections were dyed with uranium acetate and lead citrate, and observed by transmission electron microscopy (TEM).

### 2.6. Heart Isolation and Perfusion

Rats were anesthetized with sodium pentobarbital (40 mg/kg, i.p.), and hearts were quickly removed out and mounted on a Langendorff apparatus via aorta for retrograde perfusion with Krebs-Henseleit (K-H) buffer at constant pressure (10 KPa) and constant temperature (37°C). K-H buffer (in mmol/L) was composed of NaCl 118, KCl 4.7, MgSO_4_ 1.2, CaCl_2_ 2.5, KH_2_PO_4_ 1.2, NaHCO_3_ 25, and glucose 11. The buffer was saturated with 95% O_2_/5% CO_2_ (pH 7.4). A water-filled latex balloon-tipped catheter was placed into the left ventricle through the left atrium and adjusted to a left ventricular end diastolic pressure (LVEDP) of 5–10 mmHg during the initial equilibration. The distal end of the catheter was connected to a PowerLab system via a pressure transducer (model Gould P23Db, AD Instrument Ltd., Australia). After 20 min stabilization with K-H buffer, the hearts were subjected to 30 min no-flow global ischemia followed by 60 min reperfusion. Left ventricular developed pressure (LVDP), LVEDP, and the maximal differentials of LVDP (±LV*dp*/*dt*_max⁡_) were continuously recorded with PowerLab system. The data were analyzed by Chart software.

### 2.7. Measurement of the Contents of CK, CK-MB, LDH, MDA, and SOD

Enzyme linked immunosorbent assay (ELISA) kits were used to determinate the contents of Mitogen-activated protein kinases (CK), creatine kinase isoenzyme (CK-MB) and lactate dehydrogenase (LDH) in rats serum and MDA, SOD in rats left ventricular myocardium. All assays were performed according to the manufacturer's instructions. All samples and standards were prepared in triplicate.

### 2.8. Determination of Phosphorylation Degree of ERK and p38 MAPK

Western blot method was used to detect the phosphorylation degree of ERK and p38 MAPK in rats left ventricular myocardium. The protein concentration was assayed using bicinchoninic acid (BCA) method with bovine serum albumin as the standard. Then the protein was diluted to the same volume and heated at 100°C for 5 min after being added in loading buffer in proportion. The denatured protein samples were separated by SDS/polyacrylamide gel electrophoresis (SDS-PAGE) at 100 V for 2 h and transferred to polyvinylidene fluoride (PVDF) membranes. The membranes were blocked in 5% skimmed milk blocking buffer at room temperature for 1 h and then incubated overnight at 4°C with primary antibodies. After being washed with tris-buffered saline (TBS) tween for three times, the membranes were incubated with secondary antibody of horseradish peroxidase labeling goat anti-mouse IgG for 1 h at room temperature and ECL detection was used to color for 1 to 2 min. Automatically, imaging system was used for imaging and quantitative analysis; grey value which had been deducted background was obtained at last. All tests were repeated three times.

### 2.9. Statistical Analysis

Data were expressed as means ± SD. *t*-test was used to compare the data between two groups, and one-way analysis of variance (ANOVA) was used to data among multi groups. P<0.05 was considered significant.

## 3. Results

### 3.1. The Effect of Salidroside on the Myocardial Structure of Acute Exhaustive Rat

**Figure 1:**
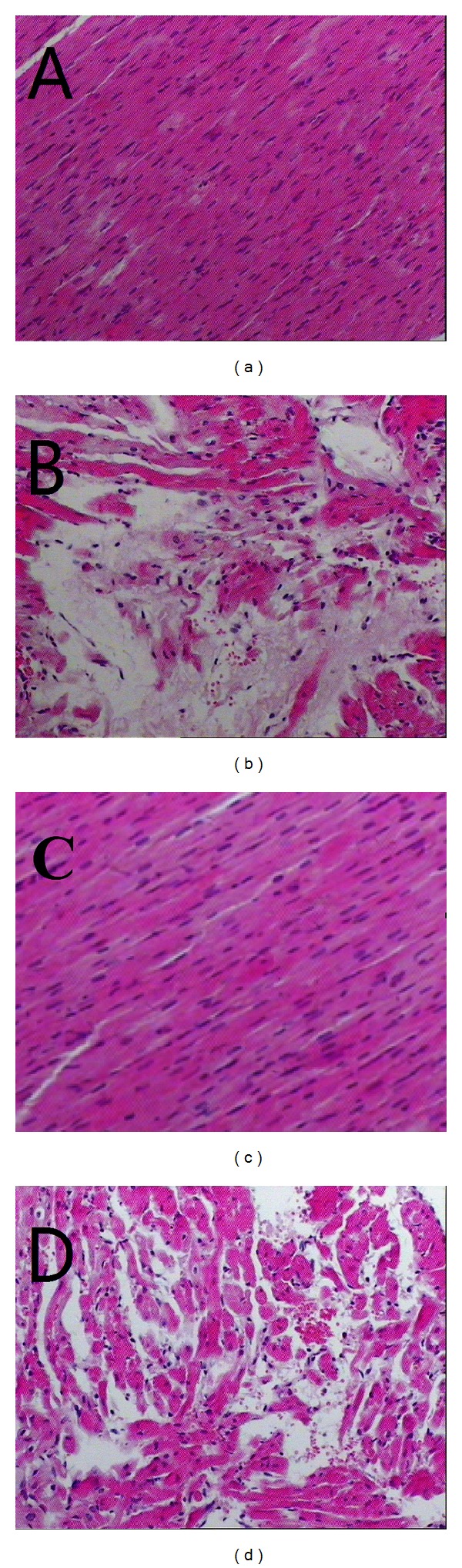
Effects of SAL on the cell morphology. Hematoxylin and eosin (HE) stain (×400). (a) Control group; (b) acute exhaustive swimming group; (c) salidroside group; (d) salidroside-acute exhaustive swimming group.

Figure 1 shows the optical microscopy of rat myocardial structure. From Figures 1(a) and 1(c) we can find that the myocardial structures of Con group and Sal group rats are as follows: muscle fibers arrange neatly, interstitial substance has no edema, muscle membrane has no damage, and muscle fibers have no fracture, degeneration, and necrosis; Figure 1(b) shows the myocardial structure of EE group rats: muscle fibers arrange irregularly, interstitial substance has edema, muscle membrane is damaged, and muscle fibers have fracture, degeneration, and necrosis. As Figure 1(d) shows, the myocardial structure of SE group rats is as follows: muscle fiber direction changes, interstitial substance has slight edema, muscle membrane has no damage, and pathological change of degeneration is visible.

**Figure 2:**
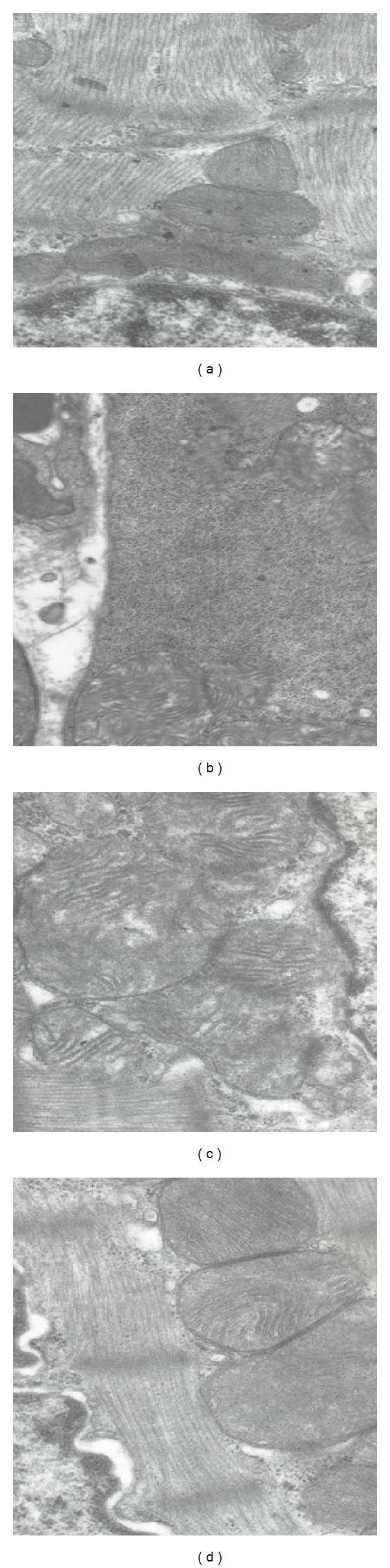
Effects of SAL on the ultrastructure of cardiomyocytes (×20,000). (a) Control group; (b) acute exhaustive swimming group; (c) salidroside group; (d) salidroside-acute exhaustive swimming group.

Figure 2 shows the transmission electron microscopy (TEM) of rat myocardial structure. Figures 2(a) and 2(c) show the myocardial structures of Con group and Sal group rats: sarcomeres arrange neatly, the density is uniform, organelles have no edema, and the membrane and crest of mitochondria are normal; Figure 2(b) shows the myocardial structures of EE group rats: myocardial nuclear matrix has edema, nuclear gap widens, the number of mitochondria and glycogen decreases significantly, the membrane and crest of mitochondria fuse partly and become blurry or missing, a small amount of muscle fiber is necrotize. As Figure 2(d) shows, the myocardial structure of SE group rats is as follows: myocardial cell matrix has edema and the membrane and crest of mitochondria fuse partly and become blurry or missing.

### 3.2. The Effect of Salidroside on the Contents of CK, CK-MB, and LDH of Acute Exhaustion Rats

**Figure 3:**
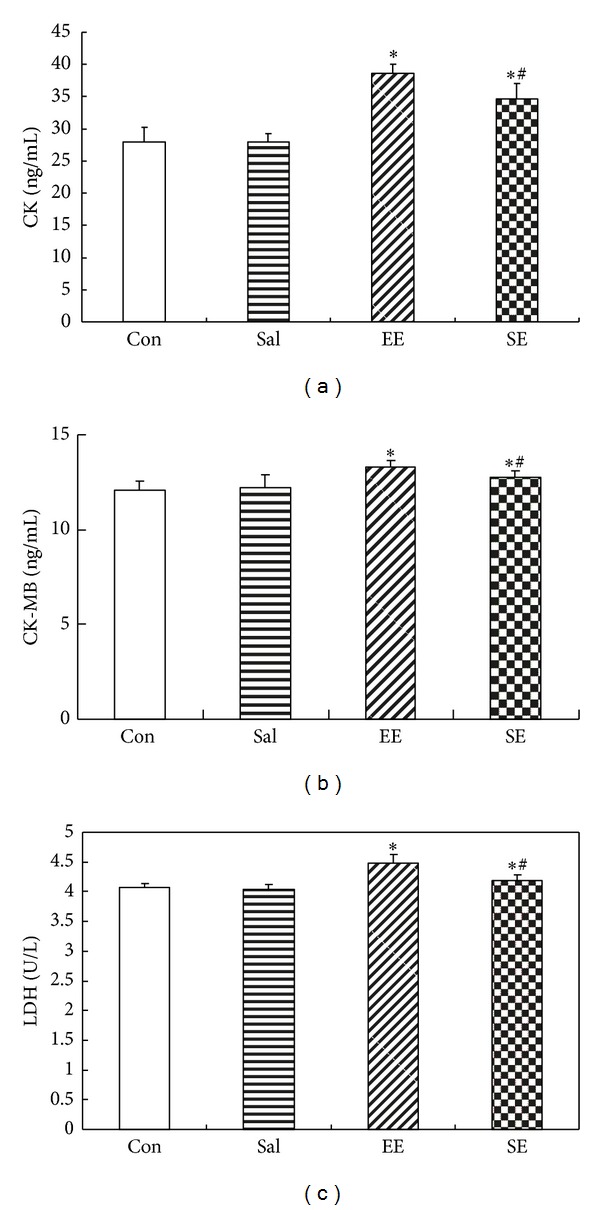
The effect of salidroside on the contents of CK (a), CK-MB (b), and LDH (c) of acute exhaustion rats in serum. *P < 0.05 versus control group; ^#^ *P* < 0.05 versus EE group. Con: control group; Sal: salidroside group; EE: acute exhaustive swimming group; SE: salidroside-acute exhaustive swimming group.

The content of serum CK (ng/mL): compared to Con group (27.990 ± 2.279), the content of serum CK in SE group (34.642 ± 2.374) and EE group (38.671 ± 1.374) increased significantly (*P* < 0.05), and in EE group it was significantly higher than in SE group (*P* < 0.05) while Sal group (27.911 ± 1.911) and Con group had no significant difference (*P* > 0.05) (Figure 3(a)).

The content of serum CK-MB (ng/mL): compared to Con group (12.104 ± 0.473), the content of serum CK-MB in SE group (12.758 ± 0.359) and EE group (13.289 ± 0.348) increased significantly (*P* < 0.05), and in EE group it was significantly higher than in SE group (*P* < 0.05) while Sal group (12.230 ± 0.637) and Con group had no significant difference (*P* > 0.05) (Figure 3(b)).

The content of serum LDH (U/L): compared to Con group (4.066 ± 0.068), the content of serum LDH in SE group (4.191 ± 0.094) and in EE group (4.474 ± 0.146) increased significantly (*P* < 0.05), and in EE group it was significantly higher than in SE group (*P* < 0.05) while Sal group (4.043 ± 0.084) and Con group had no significant difference (*P* > 0.05) (Figure 3(c)).

### 3.3. The Effect of Salidroside on the Cardiac Function of Isolated Rat Heart

Under the basic condition, compared with Con group, ±LV *dp*/*dt*_max⁡_ values of EE group significantly fell and HR sped up prominently there was significant difference between the two groups (*P* < 0.05). LVDP of EE group and LVDP of SE group were both significantly lower than that of Con group (*P* < 0.01, *P* < 0.05), and LVDP of EE group was also lower than that of SE group (*P* < 0.05). Other groups had no significant difference compared to Con group.

**Figure 4:**
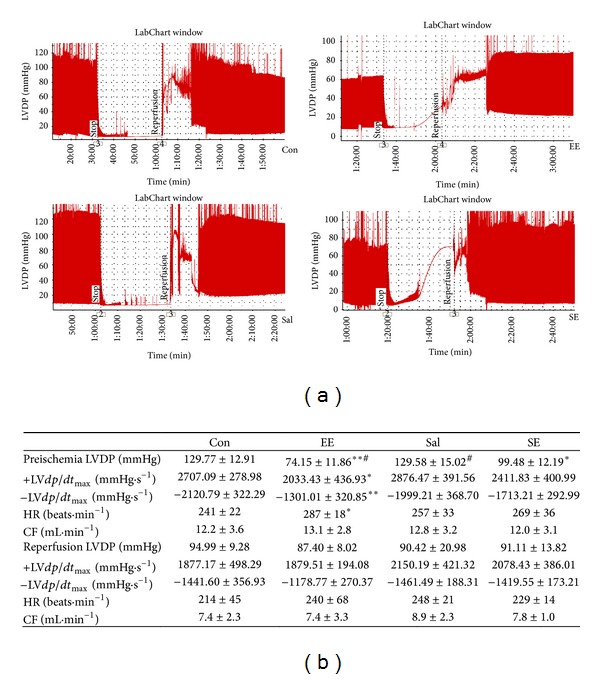
Cardiac performance in isolated rat hearts submitted to 30 min ischemia and 60 min reperfusion. (a) Original recording. (b) Cardiac functional parameters. Con: control group; EE: acute exhaustive swimming group; Sal: salidroside group; SE: salidroside-acute exhaustive swimming group. LVDP: left ventricular developped pressure; LVEDP: left ventricular end-diastolic pressure; ±LV *dp*/*dt*_max⁡_: maximum change rate of left ventricular pressure; HR: heart rate; CF: coronary flow. Data were expressed as mean ± SE, *n* = 6 for each group. *P < 0.05, **P < 0.01 versus control group; ^#^ *P* < 0.05 versus SE.

Ischemia/reperfusion after reperfusion for 60 minutes, the recovery of LVDP, +LV *dp*/*dt*_max⁡_ and − LV *dp*/*dt*_max⁡_ in Con group rats was 74%, 69% and 68%, respectively; in the Sal group rats, LVDP, +*dp*/*dt*_max⁡_ and −*dp*/*dt*_max⁡_ were recovered to 71%, 75% and 75%; the recovery of LVDP, +*dp*/*dt*_max⁡_ and −*dp*/*dt*_max⁡_ in SE group rats was 92%, 86% and 84%. And the recovery of EE group could reach to 97%, 97% and 95%. There was no significant difference between Con group and Sal group (*P* > 0.05), while the recovery of EE group and SE group was better than that of the rest of the two groups (Figure 4).

### 3.4. The Effect of Salidroside on the Contents of MDA and SOD of Acute Exhaustion Rats

The content of MDA (mmol/mg): compare with Con group (2.97 ± 0.16), the content of MDA in EE group increased significantly (*P* < 0.05). The content of SOD (ng/mg): compare with Con group (2.63 ± 0.06), the content of SOD in EE group (2.27 ± 0.13) and SE group (2.48 ± 0.08) decreased significantly (*P* < 0.05). Regarding the contents of SOD and MDA, there was significant difference between SE group and EE group (*P* < 0.05), but no significant difference between Sal group and Con group (*P* > 0.05) (Table 1).

### 3.5. The Effect of Salidroside on the Phosphorylation Degree of ERK and p38 of Acute Exhaustion Rats

The gray value ratio of p-ERK to ERK: compared to Con group (0.201 ± 0.037), the gray value ratio of p-ERK to ERK in SE group (0.967 ± 0.0788) and EE group (0.633 ± 0.087) increased significantly (*P* < 0.05), and in SE group it was significantly higher than in EE group (*P* < 0.05) while Sal group (0.229 ± 0.047) and Con group had no significant difference (*P* > 0.05).

**Figure 5:**
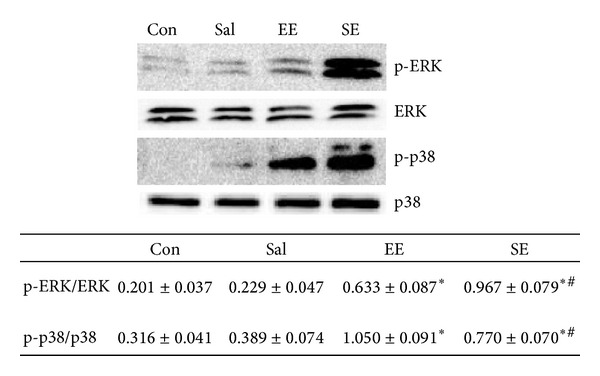
The effect of salidroside on the phosphorylation degree of p-ERK and p-p38 of acute exhaustion rats. Data were expressed as mean ± SE, *n* = 7 for each group. *P < 0.05 versus control; ^#^ *P* < 0.05 versus the acute exhaustive group. Con: control group; EE: acute exhaustive swimming group; Sal: salidroside group; SE: salidroside-acute exhaustive swimming group.

The gray value ratio of p-p38 to p38 MAPK: compared to Con group (0.316 ± 0.041), the gray value ratio of p-p38 to p38 MAPK in SE group (0.770 ± 0.070) and EE group (1.050 ± 0.091) increased significantly (*P* < 0.05), and in EE group was significant higher than SE group (*P* < 0.05) while Sal group (0.389 ± 0.074) and Con group had no significant difference (*P* > 0.05) (Figure 5).

## 4. Discussion

In this study, we mainly investigated the effect of salidroside on cardiac structure, serum myocardial enzyme, antioxidant stress, and MAPKs signal transduction of acute exhaustive exercise rats. In addition, we also observed the changes of cardiac function, heart rate, and coronary flow of isolated heart ischemia/reperfusion of acute exhaustive exercise rats.

Myocardium is one of the organizations which are sensitive to hypoxia and have high oxygen uptake. Hypoxia not only affects the ultrastructure of cardiomyocytes but also influences the metabolism and function of myocardium [17].

The TEM figures showed that the characteristics of EE group rats myocardium were myocardial nuclear matrix edema, nuclear gap widened, the number of mitochondria and glycogen decreased significantly, the membrane and crest of mitochondria were fused partly and became blurry or missing, and a small amount of muscle fiber necrosis. Acute exhaustive exercise damaged the structure of cardiomyocytes, and amounts of CK, CK-MB, and LDH were released into blood, so the contents of these enzymes in rat serum increased significantly.

The results of isolated heart ischemia/reperfusion showed, in basal state, compared with Con group, the LVESP, LVDP, +*dp*/*dt*_max⁡_, and −*dp*/*dt*_max⁡_ of EE group, that HR sped up and CF decreased. This indicated that the function of cardiac systole and diastole weakened, and exhaustive exercise had damaged the cardiac function. After ischemia/reperfusion, it caused myocardial reversible or irreversible damage; myocardial systole and diastole functions reduced further, LVEDP rose, +*dp*/*dt*_max⁡_ and −*dp*/*dt*_max⁡_ reduced, and CF decreased. This might be owed to a large number of oxygen free radicals and other products [18]. From the analysis of cardiac function, after reperfusion for 60 min, the recovery data of EE group had no significant differences with other groups, but the recovery rates of LVDP, +LV *dp*/*dt*_max⁡_, and −LV *dp*/*dt*_max⁡_ were significantly higher than those of Con group, and the recovery rates of SE group were also significantly higher than those of Con group. This might be associated with the adaption of ischemia caused by exhaustive exercise and still need further research.

Acute exhaustive exercise stimulated cardiomyocytes and caused oxidative stress; amount of ROS was produced and caused lipid peroxidation with unsaturated fatty acids on cell membrane, so a large number of MDA were generated. Moreover, the content of SOD decreased obviously because of the massive consumption of the superfluous ROS. Oxidative stress destroyed the stability of cell membrane. The liquidity is changed. The permeability is enhanced. The structure and activity of the protein are destroyed, so the structure and function of cardiomyocytes are damaged at last.

Salidroside could reduce the content of MDA and increase the content of SOD significantly. It enhanced the antioxidant system and improved the function of endogenous clearing system, which sped up the elimination of ROS, reduced the lipid peroxidation with membrane lipid, reduced the damage of ROS to cardiomyocytes, and improved the myocardium ultrastructure and cardiac function of acute exhaustive exercise to a certain extent. Furthermore, salidroside also increased the coronary flow, improved the blood and oxygen supply of ischemia area, ameliorated myocardial systole and diastole functions, and bettered the recovery of cardiac function of acute exhaustive exercise rats after ischemia/reperfusion; all of these indicated that salidroside could evidently protect the myocardium against ischemia/reperfusion injury. So, salidroside could protect the heart of acute exhaustive rats through the way of antioxidative stress.

ERK and p38 MAPK were both activated significantly in acute exhaustive rats. This might be because the ROS generated from oxidative stress caused by acute exhaustive exercise activated the access of MAPKs signaling transduction, while salidroside could inhibit the activation of p38 MAPK and promote the activation of ERK of acute exhaustive rats, which prompted that the heart protection might be related to the inhibition of cardiomyocytes apoptosis signal, the delay of cell apoptosis induced by stress. So, salidroside could protect the heart of acute exhaustive rats through the way of MAPKs signal transduction.

In conclusion, salidroside could effectively inhibit the cardiac function decreasing of acute exhaustive rats and reduce the cardiac function injury caused by acute exhaustive exercise. The protection mechanism might be associated with antioxidative stress and MAPKs signal transduction. However, we could not ensure whether salidroside has the protection against ischemia/reperfusion injury of exhaustive heart; it might be the same as the protection against myocardial ischemia caused by exhaustion and did not superimpose.

## Figures and Tables

**Table 1:** The effect of salidroside on the contents of MDA and SOD of acute exhaustion rats.

	Con	Sal	EE	SE
SOD (ng/mg)	2.63 ± 0.06	2.54 ± 0.06	2.27 ± 0.13**	2.48 ± 0.08^∗#^
MDA (nmol/mg)	2.97 ± 0.16	2.91 ± 0.19	3.34 ± 0.06**	3.17 ± 0.12^#^

Data were expressed as mean ± SE, *n* = 7 for each group. *P < 0.05 versus control group, **P < 0.01 versus control group, and ^#^ *P* < 0.05 versus the acute exhaustive group. Con: control group; EE: acute exhaustive swimming group; Sal: salidroside group; SE: salidroside-acute exhaustive swimming group.
